# Infectious Threats, the Intestinal Barrier, and Its Trojan Horse: Dysbiosis

**DOI:** 10.3389/fmicb.2019.01676

**Published:** 2019-08-07

**Authors:** Simona Iacob, Diana Gabriela Iacob

**Affiliations:** ^1^Infectious Diseases Department, “Carol Davila” University of Medicine and Pharmacy, Bucharest, Romania; ^2^National Institute of Infectious Diseases “Prof. Dr. Matei Balş”, Bucharest, Romania

**Keywords:** dysbiosis, intestinal microbiota, epigenetic modulation, short chain fatty acids, immunity, sepsis, HIV infection, liver infections

## Abstract

The ecosystem of the gut microbiota consists of diverse intestinal species with multiple metabolic and immunologic activities and it is closely connected with the intestinal epithelia and mucosal immune response, with which it builds a complex barrier against intestinal pathogenic bacteria. The microbiota ensures the integrity of the gut barrier through multiple mechanisms, either by releasing antibacterial molecules (bacteriocins) and anti-inflammatory short-chain fatty acids or by activating essential cell receptors for the immune response. Experimental studies have confirmed the role of the intestinal microbiota in the epigenetic modulation of the gut barrier through posttranslational histone modifications and regulatory mechanisms induced by epithelial miRNA in the epithelial lumen. Any quantitative or functional changes of the intestinal microbiota, referred to as dysbiosis, alter the immune response, decrease epithelial permeability and destabilize intestinal homeostasis. Consequently, the overgrowth of pathobionts (*Staphylococcus, Pseudomonas*, and Escherichia *coli*) favors intestinal translocations with Gram negative bacteria or their endotoxins and could trigger sepsis, septic shock, secondary peritonitis, or various intestinal infections. Intestinal infections also induce epithelial lesions and perpetuate the risk of bacterial translocation and dysbiosis through epithelial ischemia and pro-inflammatory cytokines. Furthermore, the decline of protective anaerobic bacteria (*Bifidobacterium* and *Lactobacillus*) and inadequate release of immune modulators (such as butyrate) affects the release of antimicrobial peptides, de-represses microbial virulence factors and alters the innate immune response. As a result, intestinal germs modulate liver pathology and represent a common etiology of infections in HIV immunosuppressed patients. Antibiotic and antiretroviral treatments also promote intestinal dysbiosis, followed by the selection of resistant germs which could later become a source of infections. The current article addresses the strong correlations between the intestinal barrier and the microbiota and discusses the role of dysbiosis in destabilizing the intestinal barrier and promoting infectious diseases.

## Introduction

The intestinal barrier defines the morpho-functional unit responsible for the defense of the intestinal mucosa and consists of the intestinal microbiota, intestinal epithelial cells (IECs) and mucosal immunity tightly linked through a complex network of cytokines, antimicrobial peptides (AMPs), metabolic products, and numerous regulatory molecules (Meng et al., 2017). Given that the intestinal mucosa is the largest body surface at risk of infectious threats, the anatomic and functional homeostasis of the intestinal barrier is a key step in the anti-infectious defense of the human organism.

The intestinal microbiota represents the first defense line of the intestinal barrier. The microbiota entails millions of microogranisms colonizing the gastrointestinal tract most of which are bacteria. This large number of microorganisms withstand the unfavorable intestinal habitat thanks to their symbiotic relationships with the human organism. These symbiotic host-commensal relationships develop after birth and enable the metabolic, immune and antiinfectious processes through which the microbiota contributes to gut homeostasis (O’Hara and Shanahan, 2006). The structural and functional stability of commensal populations is regulated through numerous signaling molecules (quorum sensing) and cellular regulators (miRNAs) as well as through other physiologic and pathologic factors. Qualitative or quantitative alterations of this microbial community broadly defined as dysbiosis impair the relationships between the host and commensal species, modify the balance between commensals and pathogens, decrease the intestinal barrier protection and favor infectious pathogens (McDonald et al., 2016). Consequently, the microbiota loses its anti-infectious role and becomes the weak link responsible for persistent infections.

The article discusses gut barrier defense mechanisms, the key anti-infectious role of microbiota inside of the gut barrier and the impact of dysbiosis in the life-threatening infections.

## The Gut Barrier and Antibacterial Defense Mechanisms

### Commensal Flora – The First Line of Defense

The intestinal microbiota encompasses all microbial species populating the gastrointestinal tract. Molecular techniques in healthy individuals have revealed a diverse ecosystem, containing nine bacterial phyla, out of which four are dominant: *Firmicutes, Bacteroidetes, Actinobacteria*, and *Proteobacteria* (Rajilić-Stojanović et al., 2007). The structure of the microbiota is gradually delineated after the age of three through symbiotic relationships with the organism and ensures the intestinal dominance of specific commensals (“symbionts”) belonging to the *Firmicutes* and *Bacteroidetes* phylum or *Bifidobacteriales* order. Symbionts will compete with pathogens or potential pathogenic germs (“pathobionts”) for pre-existent intestinal niches (“niche competition”) as well as for intestinal nutrients (“nutritive competition”). This competition ensures the structural stability of the microbiota and is referred to as “colonization resistance.” Colonization resistance employs a network of specific molecules with a critical anti-infectious roles (Sassone-Corsi and Raffatellu, 2015; Sorbara and Pamer, 2019). Among these are molecules with important metabolic and antimicrobial roles such as short chain fatty acids (SCFAs) and bacteriocins.

Short chain fatty acids are end products of the anaerobic fermentation of intestinal microbiota and the major energy source for colonocytes (Roediger, 1980). SCFAs effects are mediated by G protein-coupled receptors (GPRs) (GPR41, GPR43, and GPR109A) expressed on immune cells and a variety of tissues including gut epithelial cells. The predominant SCFAs are present at high mM levels in the colon (butyrate), entero-hepatic circulation (propionate), and systemic circulation (acetate) and are responsible for epithelial protection and regulation of the inflammatory intestinal response, reviewed in Donohoe et al. (2011) and Fukuda et al. (2011). SCFAs ensure a low antibacterial pH around colonic cells, favor mucus synthesis, and contribute to IECs integrity through the upregulation of tight junction proteins, stabilization of HIF transcription factor, IL-18 release, and NLR pyrin domain 3 (NLRP3) inflammasome modulation (Wang et al., 2012; Kelly et al., 2015; Macia et al., 2015; Feng Y. et al., 2018). At the same time SCFAs contribute to the antibacterial defense against pathogenic species through neutrophil recruitment, the release of cytokines and AMPs while also inducing the intestinal immunotolerant response against commensal species as further discussed.

Gut bacteria also, particularly *Firmicutes, Proteobacteria*, and *Bacteroidetes*, synthesize multiple microbicidal molecules (bacteriocins) with a broad spectrum of activity (Todorov et al., 2014; Drissi et al., 2015). Bacteriocin-producing strains such as lactic acid bacteria are commonly used as probiotics in the food industry (Parada et al., 2007). Certain bacteriocins (nissin, pediocin) have been approved for oral or topical use and others are studied as oral alternatives to antibiotics (reviewed in Cavera et al., 2015). Nevertheless, the oral use of bacteriocins is still uncertain due to a lack of data on their action mechanisms as well as on their efficiency, cytotoxicity, stability, and immunogenicity.

Dysbiosis modifies not only the balance between commensals and pathogens but also the release of antimicrobial molecules. In turn this disturbs the process of colonization resistance and allows the invasion of the intestinal epithelium by various pathogens.

### The Intestinal Epithelium – The Second Anti-infectious Defense Line

The intestinal epithelium has its own defense mechanisms, both structural and functional. The intestinal epithelial monolayer includes several subsets of IECs united through tight apical junctions, externally covered with a mucus layer. IECs come into direct contact with the lamina propria and immune cells (Goto and Ivanov, 2013). All intestinal epithelial lineages arise from HOPX-quiescent stem cells with immunoregulatory and tumor suppressing properties (Takeda et al., 2011). IECs include entero-absorptive enterocytes, mucus secreting goblet cells, antigen-sampling cells (M cells), and Paneth cells, each exhibiting specific surface receptors.

The IECs express specific receptors namely “pattern recognition receptors” (PRRs) such as Nod-like receptors (NLRs), toll-like receptors (TLRs) and other PRR families located on the cell membrane or in the cytoplasm which recognize specific microbial-associated molecular patterns (MAMP). Once the activation of these receptors induces cytoplasmic signal transduction cascades and further promotes the NF-kappaB (NF-kB) pathway along with other cellular transcription factors, inflammasome activation and pro-inflammatory cytokines (IL-17, IL-18, IL-22) (Hirota et al., 2011; Jones and Neish, 2011; Levy et al., 2015). The IECs and particularly Paneth cells residing at the bottom of the intestinal crypts release AMPs (human-cathelicidin, defensins) with microbicidal, immunomodulatory and wound-healing properties. Thus IECs further contribute along with microbial colonization resistance to the stabilization of the gut barrier. Therefore Paneth cell MyD88 expression is an essential mechanism for the restriction of intestinal translocation and penetration by enteric commensals or pathogens (Vaishnava et al., 2008).

Short chain fatty acids and GPR43/109A stimulation also protects epithelial integrity via inflammasome activation and epigenetic immunomodulation of FoxP3+ regulatory T cells (Treg) proliferation as shown below (Macia et al., 2015).

Dysbiosis induced by infectious or non-infectious causes (ischemia, inflammation, tumors, or various treatments) favors the distruption of the intestinal epitelium (Kitajima et al., 1999; Nagpal and Yadav, 2017; Stewart A. S. et al., 2017). The injured epithelium permits the translocation of various intestinal bacteria and of their toxins including endotoxins (lipopolysaccharide, LPS). In turn these translocations lead to bacteraemia, endotoxemia and life-threatening infections (sepsis, primitive peritonitis, portal encephalopathy in cirrhotic patients, or immune activation in human immunodeficiency virus (HIV) infection.

### The Intestinal Immunity – The Third Anti-infectious Defense Line

The intestinal epithelium represents the largest epithelial surface in the human body. It connects with the intestinal ecosystem containing commensal and pathogenic species. Commensal germs need to be immunologically accepted, whereas intestinal pathogens must be eliminated. This differentiation requires the activation of an extremely well-coordinated and efficient immune system. Considering that intestinal commensals are present in the human body ever since birth they represent, in fact, the first contact between the immune system and the exterior environment. As such, this large and diverse microbiota will serve as one of the body’s defense mechanisms against the invasion of pathogenic germs. At the same time, the microbiota develops mutually beneficial relationships with the organism and together with the intestinal epithelium and gut-associated lymphoid tissue (GALT) the microbiota forms a complex intestinal barrier against infectious threats.

These symbiotic relationships are facilitated by the presence of PRRs and by the immunomodulatory capacity of the intestinal microbiota against the antigenic structures exposed by the intestinal microbiota.

#### The Role of Cellular Receptors

The intestinal epithelium expresses numerous types of receptors able to recognize different MAMPs expressed by microbial species and to convert them into gut signals for inflammatory cascades (Kim et al., 2004). Thus TLR 1,2,4,5,6 (extracellular sensors) and NLR1,2 and TLR9 (cytoplasmic sensors) are expressed on epithelial cells and act complementary, promoting both innate and adaptive immunity (Kim et al., 2004; Abreu et al., 2005). MAMPs are expressed by commensal species since birth and examples of MAMPs include the LPS found in the outer membrane of Gram-negative bacteria, lipoteichoic acid present on the gram positive bacteria wall, peptidoglycan, a component of the bacterial wall, flagellin, a component of intestinal flagellated bacteria, or release outer membrane vesicles that function as PRR ligands (Cañas et al., 2018). PRRs activation in the presence of MAMPs stimulates the NF-kB signal transduction pathway, induces pro-inflammatory interleukins and enables an innate immune response lastly maintaining a state of controlled inflammation (Hayashi et al., 2001; Bambou et al., 2004; Vora et al., 2004; Rhayat et al., 2019). In the absence of MAMPs or of specific receptors (germ free animals or genetic mutations of receptors) the organism fails to detect microbial antigens and does not mount adequate defense mechanisms (Abreu et al., 2005). Hence, it is considered that the rich and diverse commensal flora plays an active role in the proper development of the immune system since birth.

Antigen-presenting cells (APC) belonging to GALT such as macrophages and dendritic cells (DCs) also exhibit cell receptors which regulate numerous genes and modulate the release of NF-κB transcription factor, immunomodulatory cytokines and AMP. Depending on the type of activated receptor DCs may generate a Th1/Th17 pro-inflammatory response. Thus, to exemplify, the NLR2 induced by commensal bacteria appears to play a central role in the downregulation of the GALT inflammatory activity and the ability of DCs to induce a polarized CD4^+^Th1 response in mice and human experiments (Butler et al., 2007; Barreau et al., 2010).

By activating the IECs or APCs receptors, the commensal flora induces a tolerogenic response in DCs, characterized by the release of immunosuppressive cytokines (IL10, IL4, TGF-β), promotion of Treg cells and of CD4^+^Th2 phenotype in the periphery (Iwasaki and Kelsall, 1999; Stagg et al., 2003; Atarashi et al., 2013; Maharshak et al., 2013; Martin-Gallausiaux et al., 2018). The induction of mucosal immune tolerance protects the epithelia from a detrimental inflammatory immune response and contributes to the immune system’s maturation (Jung et al., 2019). Consequently any disturbance involving this process plays an essential role in intestinal inflammatory processes.

The activation of cellular receptors is intricately linked to the pro-inflammatory response of mesenteric DCs and is particularly involved in the maintenance of the Th17/Th1 response directed against pathogens (reviewed in Sorini et al., 2018). What is interesting is that DCs may activate Treg cells to certain antigens such as LPS of the commensal flora but not LPS of the pathogenic flora (Shirai et al., 2004). Thus the stimulation of mice with *E. coli*-derived LPS activates inflammatory mechanisms (IL-12 production, Th1 response) whereas the stimulation with LPS derived from *Poprhyromonas gingivalis* induces IL-4 production and a Th2 response (Pulendran et al., 2001). Similarly TLR recognize flagellin antigens of pathogenic Salmonella species and trigger an inflammatory response against them, while commensal species lacking this antigen do not induce an inflammatory response (Gewirtz et al., 2001).

All these aspects evince that cell receptors represent a filter for intestinal signals and play a deciding role in the induction of specific defense mechanisms adapted to the commensal flora. This role of cell receptors is indeed significant considering the density and variety of the intestinal bacteria. Dysbsiosis eliminates commensal species that trigger Treg-cell polarization and therefore leads to an excessive Th1 or Th2 response, further promoting an inflammatory or autoimmune response.

#### The Immunomodulatory Role of GALT

Gut-associated lymphoid tissue is the most important lymphatic network in humans and involves isolated or aggregated lymphoid follicles (Peyer’s Patches), intra-epithelial lymphocytes, macrophages, DCs, mesenteric ganglia, secretory IgA (sIgA) cells and lymphatics.

Peyer’s patches localized in the mucosa and submucosa of the small intestine are covered by a “follicle-associated epithelium” containing specialized “M cells”. These cells engulf and transport antigens from the intestinal lumen to intestinal DCs where T cell lymphocytes (LT) are primed (Alpan et al., 2001). LT subsequently return to the intestinal lymphatic compartment for their effector function. At the same time, M cells initiate mucosal sIgA production and humoral responses (Rios et al., 2016). Thus, Peyer’s patches directly mediate the interaction between the intestinal flora and the humoral or cellular immune response. Enteric pathogens are adapted to invade and destroy follicle-associated epithelium, especially M cells, further interfering with T-cell differentiation and the immune response; these germs adhere to the intestinal epithelium through different mechanisms (Phillips et al., 2000), invade the enterocytes (Kühbacher et al., 2018), or invade and destroy M cells (Clark et al., 1994; Autenrieth and Firsching, 1996; Penheiter et al., 1997; Corr et al., 2006).

Gut-associated lymphoid tissue also harbors a tolerogenic DCs population namely CD103^+^DCs involved in Foxp3 expression and intestinal conversion of naïve CD4^+^T cells into Treg cells. Commensal flora stimulates CD103^+^ DCs through the activation of specific receptors or through its own metabolic products such as SCFAs (Furusawa et al., 2013; Nastasi et al., 2015; Kaisar et al., 2017). Thus, butyrate stimulates intestinal DCs, ensures intestinal immune tolerance through IL-10 release (Liu et al., 2012) and T cell polarization toward Treg cells while maintaining the balance between the immunosuppressive IL-10-secreting CD4^+^T cells, IL-17-secreting Th17 cells, and the CD4^+^Th1 effector cells (Arpaia et al., 2013; Furusawa et al., 2013). Additionally, SCFAs attenuate the excessive inflammatory response induced by LPS-producing Gram negative bacteria (Cox et al., 2009).

The dysregulation of the Th1/Th17 immune response against commensal species is associated with intestinal inflammatory diseases. On the other hand the excessive polarization toward a Treg response attenuates the immune response to infections (Brenchley et al., 2004). Another example of GALT dysregulation occurs in HIV due to the destruction of the intestinal gut barrier and the ensuing chronic inflammatory response. GALT depends on the microbiota and it is worth noting that the development of GALT and the activation of T cell lymphocytes and B cell lymphocytes cannot occur in the absence of signals released by the intestinal flora.

Experiments on commensals belonging to *Clostridium* and *Bacteroides* species further highlighted the ability of the intestinal microbiota to maintain the intestinal homeostasis and to orchestrate an adequate T-cell response through specific MAMPs during bacterial invasion (Ivanov et al., 2008; Lécuyer et al., 2014). Thus, *Bacteroides fragilis* strains expressing polysaccharide A binding TLR2 on CD4^+^T cells and gut-indigenous *Clostridium* belonging to XIVa and IV clusters favor Tregs, suppress Th-17 cells and facilitate mucosal tolerance toward the colon microbiota (Mazmanian et al., 2008; Round et al., 2011; Atarashi et al., 2013). On the other hand segmented filamentous bacteria, a genetic relative of the genus *Clostridium* promote both effector Th17 CD4+T cells (Ivanov et al., 2009; Farkas et al., 2015; Schnupf et al., 2017) and sIgA antibodies during intestinal invasion (Lécuyer et al., 2014). Other microbial species also modulate the Treg/Th17 axis, potentially controlling the intestinal inflammation and tolerance (Pandiyan et al., 2019). This aspect also explains the disequilibrium between these species during dysbiosis as well as the expansion of pathogens and of mucous lesions.

#### Epigenetic Regulatory Implications

Modulation of gene expression by epigenetic mechanisms in the intestinal environment was studied especially for neoplasms and inflammatory processes (Vdovikova et al., 2018). Epigenetic mechanisms modulate gene transcription by various processes: DNA methylation, histone modifications and modulation of long non-coding RNA and microRNA expression. However the mechanisms through which bacteria are either affected or induce gut epigenetic changes are not well understood.

Histone modifications are correlated with the activation or repression of genetic transcription through the modulation of two antagonistic enzymes, namely histone acetyl transferases (HATs) inducing histone acetylation, and histone deacetylases (HDACs) inducing histone deacetylation. During acetylation, the chromatin structure loosens and can be accessed by transcription factors. HDAC inhibitors increase histone acetylation and subsequently regulate gene expression in numerous immune cells such as epithelial cells, neutrophils, APCs and T cells. On the contrary, deacetylation of histones by HDACs prevents gene transcription. Thus studies performed *in vitro* showed that HDAC inhibitors promote the release of several transcription factors such is NFκB, MyoD, p53, or HSP90 (Vinolo et al., 2011; Kumar S. A. et al., 2018; Banik et al., 2019). The NF-kB imbalance has been correlated with numerous inflammatory and antiapoptotic mechanisms that interfere with viral evasion (Le Negrate, 2012; Carrasco Pro et al., 2018), septic shock (Liu et al., 1999; Han et al., 2002), or inflammatory diseases (Makarov, 2001; Lawrence, 2009). Likewise, p53 regulates the cell cycle and apoptosis, functioning as a tumor suppressor (Aubrey et al., 2018). P53-mediated apoptosis was associated with the spread of viral infections (Lazo and Santos, 2011; Aloni-Grinstein et al., 2018), while HSP90 reverse transcriptase mediated activity was, most likely, associated with extensive hepatitis B virus (HBV) infection (Hu and Seeger, 1996). Several viruses among which HIV, HBV, hepatitis C virus, Epstein Barr virus, cytomegalovirus, herpes simplex virus and human T-lymphotropic virus have NF-kB activation strategies and cell apoptosis blockage (Santoro et al., 2003) while others, especially the oncogenic ones have p53 suppression mechanisms (Sato and Tsurumi, 2013). It has been demonstrated that HDAC inhibitors could block stellate cell activation thus hindering liver fibrosis in experimental animal models (Park et al., 2014; Ding et al., 2018).

By inhibiting HDAC, SCFAs could therefore control numerous infectious and immune processes (Vinolo et al., 2011; Zhou et al., 2017b; Sun et al., 2018). Although the role of HDAC inhibitors hasn’t been completely elucidated yet, most experimental studies evinced an anti-inflammatory dose dependent effect (Yin et al., 2001; Le Poul et al., 2003; Weber and Kerr, 2006; Asarat et al., 2016; Kaisar et al., 2017; Li et al., 2018) and an immunosuppressive role of SCFAs in tumors (Villagra et al., 2010; Tang et al., 2011). Still, while other HDAC inhibitors are already used in oncologic therapies, the exact role of SCFAs in the immune and tumor processes remains purely theoretical (Meijer et al., 2010; Matthews et al., 2012; Ulven, 2012).

MicroRNAs (miRNAs) are small, evolutionary conserved non-coding RNAs of approximately 19-23 nucleotides involved in the post-transcriptional regulation of cellular mRNAs. The biogenesis of mature miRNA includes a two-step cleavage process from primary miRNAs (pri-miRNA). The mature miRNA is then loaded into the effector complex RNA-induced complex (RISC). RISC interacts with target mRNA and induce mRNA cleavage or translational repression hence controlling diverse metabolic or cellular pathways including cell cycle progression, differentiation, apoptosis, immune regulation or oncogenesis (Dalmasso et al., 2011; Singh et al., 2012; Nakata et al., 2017). miRNAs are released extracellularly by most eukaryotic cells and various types of small non-coding RNAs (sRNAs) around 50–200 nt in length have been observed within extracellular vesicles released by gram negative bacteria (“outer membrane vesicles”) (Gong et al., 2011). The general conception is that sRNA, as well as microRNA-size small RNAs (msRNAs) of 15–25 nucleotides in length such as msRNA observed in the model bacterium *E. coli* (Kang et al., 2013) act as post-transcriptional regulators and function as signaling molecules for bacterial growth and virulence under experimental conditions (Ortega et al., 2012; Zhao et al., 2017) and virulence mechanisms, at least under experimental conditions (Padalon-Brauch et al., 2008; Choi et al., 2017). The role of these structures in intestinal homeostasis, has been, for now, scarcely covered, most information having been acquired from studies on conventional or germ-free mice.

Intestinal miRNAs are released by IECs (Liu et al., 2016) and are regulated by intestinal microbiota through TLR/MyD88-dependent pathway (Dalmasso et al., 2011; Singh et al., 2012; reviewed in Eulalio et al., 2012; Williams et al., 2017). Liu and Weiner (2016) conducted an extensive study identifying numerous types of extracellular miRNAs circulating in exosomes in the gut lumen and feces of mice and humans. The study proved that IECs and Hopx-expressing cells are the main sources of exosomal intestinal miRNAs and also highlighted their uptake by intestinal bacteria and potential role in post-transcriptional regulation of bacterial genes.

Studies on mice indicate a certain pattern of miRNA gut compartimentalization after bacterial colonization (Dalmasso et al., 2011) and reported the dominance of miR-143,-145 in the jejunum and cecum and of miR-200b in the large intestine and caecum (Singh et al., 2012). On the other hand the human cells or animal experimental models with pathogenic species (*Helicobacter pylori, Citrobacter rodentium, Listeria monocytogenes, Francisella tularensis*, and *Salmonella enterica*) induce a different miRNA panel mainly represented by miR-155 and miR-146 (Eulalio et al., 2012; Archambaud et al., 2013; Staedel and Darfeuille, 2013).

On a molecular level, there is a growing interest for the intestinal role of miRNAs and their regulatory implications in the gut-barrier functionality but data on this topic is divergent, scarce and fragmented. Cellular and extracellular miRNAs from the intestinal lumen modulate the epithelial integrity, inflammatory response, and probably bacterial gene mRNAs through insufficiently known mechanisms (reviewed in Belcheva, 2017).

Studies on human intestinal cells have documented the miRNA importance in the protection of epithelial tight junctions (e.g., miRNA-122) (Ye et al., 2011), epithelial regeneration (e.g., miR-143, miR-145) (Chivukula et al., 2014), and proliferation (miR-30 family members) (Peck et al., 2016), the modulation of epithelial integrity (e.g., miR-122) (Ye et al., 2011), mucin gene expression (Mo et al., 2016), and epithelial permeability (e.g., miR-21-5p) (Nakata et al., 2017).

Intestinal miRNAs are also key regulators of the immune response against infections. Host miRNAs orchestrate the immune response through PRR families and TLR signaling pathways (miR-146a) (Xue et al., 2011, 2014), while the microbiota downregulates the expression of miR-10a and miR-107 in host DCs, decreases the release of proinflammatory cytokines in mice and controls the excessive inflammatory response in human and mice (e.g., miRNA-146a and miR-193a-3p) (Taganov et al., 2006; Nahid et al., 2011; Singh et al., 2012; Dai et al., 2015). MiRNAs could also potentially influence the microbiota and alleviate colonic inflammation as was suggested by a negative correlation on ulcerative colitis between the release of miR-193a-3p and colonic inflammation (Dai et al., 2015). This concept was further explored in a study in which the oral ingestion of endotoxin in mice led to the upregulation of epithelial miR-146 and promoted innate immune tolerance and epithelial protection in the postnatal period (Chassin et al., 2010). On a similar note mi-RNA-155 defective mice failed to develop a protective immunity toward *H. pylori* or *Salmonella* Typhimurium (Rodriguez et al., 2007).

Pathogenic bacteria also release membrane or outer-membrane vesicles containing sRNAs that modulate the host miRNA profile and gene expression (Gu et al., 2017). Some studies on intestinal inflammation due to enteropathogens revealed the upregulation of miR-16,-21,-223,-594 and miRNA-31 or downregulation of miR-124 within the human intestinal lumen (Wu et al., 2010; Koukos et al., 2013; Lin et al., 2014). Their biological relevance is under study.

Various miRNAs have been correlated with intestinal oncogenesis according to human studies. In this respect miR-182,-503 and miR-17∼92 clusters modulate glycan production and correlate with the growth of certain bacteria which could potentially initiate the microenvironmental changes in colorectal cancer (Yuan et al., 2018). Gut miRNAs could therefore induce and perpetuate dysbiosis favoring various infections or intestinal cancers (Liu et al., 2016; Williams et al., 2017). Furthermore, single nucleotide polymorphisms of miRNA-146a,-27a genes promote various infections associated with inflammatory and neoplasic intestinal changes (Song et al., 2013; Shao et al., 2014). Butyrate was also shown to affect miRNA-106b expression in IECs and to modulate carcinogenesis (Hu et al., 2011). Therefore, it is probable that epigenetic regulators such as miRNAs could play a key role in the interaction between host and microbiota and miRNA dysregulations.

## Dysbiosis, the Trojan Horse Inside the Gut Barrier

### Dysbiosis and Enteral Infections

Enteric pathogens alter the intestinal barrier, antagonize the intestinal microbiota, and trigger enteral infections through various mechanisms including increased intestinal inflammation, the release of bacteriocins and upregulation of AMPs and toxin delivery secretory systems (T6SS, T3SS) as well as the exploitation of nutrients or intestinal niches (reviewed in Rolhion and Chassaing, 2016; Sorbara and Pamer, 2018).

Dysbiosis occurs as a consequence of different enteroinvasive or entorotoxinogenic species which distrupt colonization resistance, reduce the protective species belonging to the *Bacteroidetes* and *Firmicutes* phylum (especially the *Clostridiales incertae sedis* XI or the IV/XIVa cluster) and allow the overgrowth of species belonging to the *Proteobacteria* phylum (especially *Enterococcaceae* and *Enterobacteriaceae* families) (Vincent et al., 2013; Livanos et al., 2018). Antibiotic treatment of intestinal infections additionally modifies the structure of the microbiota and drastically disturbs the process of “colonization resistance” (Panda et al., 2014).

As a result, the first alterations due to dysbiosis include the loss of commensals synthesizing bacteriocins and SCFAs such as *Bacteroides thuringiensis* and the *Lachnospiraceae* and *Ruminococcaceae* families (Rea et al., 2010) along with the loss of *Clostridia* commensals competing with pathobionts for the same intestinal niche (Sailhamer et al., 2009; Buffie et al., 2015; Geerlings et al., 2018) and of *Bacteroides thetaiotaomicron* and *Akkermansia muciniphila* which protect the gut barrier (Hooper et al., 2001; Donato et al., 2010; Martín et al., 2019). In these cases, the normally scarce pathobionts eventually become the dominant species and display various virulence mechanisms. Consequently, pathobionts or their endotoxins trigger intestinal infections, gut sepsis or postantibiotic colitis (Brown et al., 2013; Pérez-Cobas et al., 2013). Moreover, antibiotic treatment favors the development of multidrug-resistance species (MDR) and in turn these become invasive and pathogenic after extraluminal translocation (Hirakata et al., 2002; Ayres et al., 2012). Hence, *Pseudomonas* MDR species with efflux pump systems like MexAB-OprM develop quorum sensing machinery to sense host stress and express multiple virulence determinants (Hirakata et al., 2002). The wide distribution of efflux pump systems in MDR intestinal species after antibiotic treatment increases the virulence of these species and their associated risk of extraluminal translocation (Nishino et al., 2006; also reviewed in Nikaido, 1996). Therefore dysbiosis may be both the cause and the consequence of enteral infection.

The restoration of the microbiota is particularly difficult and is best ensured through fecal microbiota transplantation (FMT) from a healthy donor. The effectiveness of FMT in the management of *C. difficile* enterocolitis or sepsis dysbiosis further underlines the critical role of the microbiota for gut health (Gough et al., 2011; Rao and Young, 2015; Wei et al., 2016).

### Dysbiosis and Sepsis

Sepsis is a life-threatening organ dysfunction caused by a dysregulated inflammatory response to infectious agents or their proinflamatory products. Sepsis has a progressive and fatal course to generalized microvascular injury, cellular hypoxia, and shock (Singer et al., 2016). Under certain conditions enteric bacteria could elicit a dysregulated systemic inflammatory response causing sepsis (Deitch et al., 1994; Mainous et al., 1995). The germs most commonly encountered in gut-derived sepsis are Gram negative bacilli producing either endotoxins or pore-forming exotoxins (Wallace et al., 2000) and probably one of the most common species involved in these cases is *E. coli*, a common gut resident (MacFie et al., 1999). The presence of pore-forming exotoxins is followed by the efflux of cellular potassium and activation of NLRP3 inflammasome. In turn these contribute to cellular apoptosis and loss of epithelial integrity and permit the access of toxins and pathogens to the portal and systemic circulation. This further stimulates the releases of various pro-inflammatory and blood coagulation mediators and a cytokine storm followed by vasoconstriction, ischemia and peripheral necrosis and finally organ dysfunction and shock (reviewed in Los et al., 2013; Sonnen and Henneke, 2013). The excessive activation of the CD14/TLR4/MD2 complex by bacterial endotoxins also induces the inflammasome signaling pathway, along with caspase activation (Hotchkiss et al., 1999), cells lysis and disproportionate release of pro-inflammatory cytokines, further eliciting and exacerbating sepsis, reviewed in Lamkanfi (2011), Gao et al. (2018), and Skirecki and Cavaillon (2019).

Sepsis alters the intestinal barrier through multiple factors which ultimately promote dysbiosis including intestinal ischemia and inflammatory lesions, aggressive care, antibiotic treatments, intestinal comorbidities, parenteral nutrition, etc. (reviewed in Hassoun et al., 2001; Alverdy and Luo, 2017; Fay et al., 2017). Gut vascular dysfunctions in sepsis and particularly intestinal ischemia stimulates pro-inflammatory cytokines, activates HIF-1 alpha factor, disrupts the epithelial tight junction and finally induces colonic cell apoptosis (Hassoun et al., 2001; Li et al., 2009; Yoseph et al., 2016). All of these events increase the permeability of intestinal epithelia and promote intestinal translocations (Diebel et al., 2003). The intestinal translocation of bacteria or microbial products such as endotoxin-LPS or MAMPs perpetuates and aggravates the systemic pro-inflammatory response further speeding the progression to organ failure and death (Shimizu et al., 2006, 2011; also reviewed in Deitch and Berg, 1987; Doig et al., 1998; Meng et al., 2017).

Dysbiosis entails the decrease of anaerobes *Bifidobacterium* and *Lactobacillus* (the main producers of SCFAs) accompanied by a detrimental increase of intestinal pathobionts such as *E. coli* or *Staphylococcus* and *Pseudomonas* species (Shimizu et al., 2006, 2011; Hayakawa et al., 2011; Ayres et al., 2012) replacing the protective microbiome. Additionally, the reduction of SCFAs favor the cascade release of LPS-induced pro-inflammatory mediators, LPS-triggered macrophage migration and massive release of nitric oxide by neutrophils (PMN), thus promoting intestinal inflammation and destabilizing the gut-barrier (Maa et al., 2010; Wang et al., 2017). Certain strains or specific phylogenetic groups of intestinal Gram negative bacilli (*E. coli* and *Pseudomonas aeruginosa*) acquire additional virulence genes during colonization or even change their morphotype after translocation to express virulence genes and to avoid host defense mechanisms giving rise to gut-derived sepsis (Johnson et al., 2001; Zaborina et al., 2007; Hickey et al., 2018). Thus, dysbiosis induces and supports sepsis associated mechanisms. In favor of this hypothesis, Souza et al. (2004) administered LPS to germ free mice and reported the absence of a severe inflammatory response and low mortality. Literature data on sepsis dysbiosis in newborns has confirmed significant changes of the microbiome, a decreased bacterial diversity, pre-sepsis gut colonization with invasive species (Carl et al., 2014), intestinal translocations (Madan et al., 2012; Mai et al., 2013; Taft et al., 2015; Stewart C. J. et al., 2017) or the activation of virulence and antibiotic resistance factors in intestinal germs (Mittal and Coopersmith, 2014). Of note, these alterations continue to occur throughout the progression of sepsis.

The administration of probiotics in sepsis (*Lactobacillus rhamnosus* and *Bifidobacterium longum*) decreases epithelial apoptosis as well as the release of cytokines and bacterial translocations in experimental mice models (Khailova et al., 2013; Panpetch et al., 2017). Probiotics can also restore the lactic acid-producing flora and ensure colonization resistance toward pathogenic flora. According to Haak and Wiersinga (2017), probiotics further influence the prognosis in sepsis by down-regulating colonic TLR-2/TLR-4 via MyD88 and by mitigating the systemic pro-inflammatory response. However, their benefit to reduce the probability of sepsis in critically ill patients is questionable (Jain et al., 2004; Kotzampassi et al., 2006; Jacobi et al., 2011; Panigrahi et al., 2017). On the other hand, data on prebiotic supplementation in infections is discordant and clear recommendations are missing (Srinivasjois et al., 2013; Chi et al., 2019).

### Dysbiosis and Liver Infections

The liver is connected to the intestine through the portal vein, biliary tract, and numerous signaling molecules, together building the “gut-liver axis.” As a result, the liver is continuously exposed to microbial and metabolic molecules produced by intestinal bacteria (SCFAs, MAMPs) and further releases its own antibacterial products (bile salts) involved in the intestinal homeostasis (reviewed in Macpherson et al., 2016). Thus intestinal MAMPs reach the portal system and hepatic sinusoids and are further processed by Kuppfer cells and liver-resident T cells. SCFAs released by the microbiota are absorbed in the colon and filtered intrahepatically where most modulate the immune response, oncogenesis and are involved in the epigenetic control of liver pathogens through HDAC inhibition and transcriptional changes (Candido et al., 1978). As HDAC inhibitors, SCFAs decrease HBV replication (Pollicino et al., 2006), mediate hepatitis C virus replication (Taguwa et al., 2008, 2009), impede liver necrosis and prevent hepatocellular carcinoma, reviewed in Koumbi and Karayiannis (2015). Recently, butyrate was shown to regulate the expression of tumor-suppresive miRNAs (miRNA-26a,-26a-1,-192, etc.) through the bile acid nuclear receptor, farnesoid X receptor (FXR) while also promoting hepatocyte apoptosis through miR-22 up-regulation in hepatic cells (Pant et al., 2017; reviewed in Feng Q. et al., 2018).

Host liver miRNAs are able to regulate the replication of viral hepatitis yet the precise roles of intestinal miRNAs in the regulation of hepatotropic viruses remain unclear (Bandiera et al., 2016; Feng Q. et al., 2018; reviewed in Fan and Tang, 2014; Li et al., 2016). Hepatocytes also release immunoregulatory molecules such as primary bile acids (BA). A small proportion of primary BA are dehydroxylated in the intestine through bile salt hydrolases and converted to secondary BA entering the entero-hepatic circulation. The latter exert a bacteriostatic effect on the intestinal barrier and an anti-fibrotic and anti-inflammatory intrahepatic activity (Wu et al., 2012).

Dysbiosis is closely related to liver pathology. The liver provides an immunotolerogenic environment with an attenuated immune responsiveness and an increased risk of persistent infections (Crispe, 2014). Chronic liver diseases and their neurologic complications (hepatic encephalopathy and coma) are induced and aggravated by changes of the gut microbiota such as the overgrowth of pathobionts (*Enterobacteriaceae, Bacteroidaceae*, and *Enterococcus* species) and the dramatic decline of commensals such as *Roseburia*, *Bifidobacterium*, and *Lactobacillales* (Wu et al., 2012; Bajaj et al., 2014; Schnabl and Brenner, 2014). On the other hand cirrhosis modifies the composition of the microbiota, namely it decreases commensal *Clostridia* species (the clusters XI and XIVab), the *Bacteroidales-Prevotella* group and butyrate-producing *Roseburia* genus (Lu et al., 2011; Bajaj et al., 2012; also reviewed in Schnabl, 2013; Kang and Cai, 2017). HBV associated cirrhosis significantly reduces the *Bifidobacterium/Enterobacteriaceae* ratio and increases the bacterial virulence through adhesins and toxins (Lu et al., 2011). The ensuing dysbiosis compromises the integrity of the intestinal barrier and favors bacterial translocations, bacteraemia, or endotoxemia (Mainous et al., 1991; Lin et al., 1995; Bajaj et al., 2014). Given the reduced phagocytosis and canalization of porto-systemic shunts in this setting, bacterial translocations increase the risk of primitive peritonitis and septic shock.

Translocated bacteria and their derived products enter the portal circulation and liver sinusoids, promote liver inflammation (Seki et al., 2007) and aggravate the fibrogenic and oncogenic potential of liver diseases, reviewed in Yang and Seki (2012).

The mechanisms through which intestinal dysbiosis promotes liver injury employ the activation of TLR signaling pathways, reviewed in Seki and Schnabl (2012). Hence, LPS binding to TLR4 receptors of Kuppfer cells triggers a cascade of inflammatory cytokines (Su et al., 2000) and plays a key role in hepatic injury. Bacterial DNA activates TLR9 signaling pathways in Kuppfer and stellate cells which further induces liver inflammation and fibrogenesis in animal models (Miura et al., 2010). NLRP3 and NLRP6 inflammasome deficiency due to intestinal dysbiosis activate TLR4 and TLR9 pathways and exacerbate hepatic inflammation (Henao-Mejia et al., 2012). Therefore dysbiosis ensures a pro-inflammatory intrahepatic environment (Richer et al., 2013; Miura and Ohnishi, 2014; Carrasco Pro et al., 2018) and supports the progression to liver cirrhosis. The imbalance of BA synthesis in dysbiosis contributes to liver inflammation, fibrosis and carcinogenesis as well as to small intestine bacterial overgrowth, which in turn prompts an intestinal inflammatory response (Gunnarsdottir et al., 2003; Jun et al., 2010; Ridlon et al., 2014).

Considering the pathogenic role of microbiota in liver diseases various authors investigated therapeutic and prophylactic interventions. Studies on the latter explored the therapeutic use of probiotics (mostly *Lactobacillus* and *Bifidobacterium* species), antibiotics (Rifaximine), gut-derived hormones and even FMT for the manipulation of the gut-liver axis (Ponziani et al., 2015; Zhou et al., 2017a; Román et al., 2019; also reviewed in Wiest et al., 2017). Concurrently, the administration of conjugated BA in rats could normalize bile secretion and reduce intestinal bacterial overgrowth and translocations (Lorenzo-Zúñiga et al., 2003).

### Dysbiosis and HIV Infection

Human immunodeficiency virus is a retrovirus with CD4^+^T cell tropism leading to a severe immunodeficiency and AIDS, followed by secondary infections, reactivations of opportunistic germs and HIV-associated cancers. HIV induces systemic immune activation, alters the intestinal barrier and promotes irreversible metabolic, cardiovascular and neurologic changes (Steele et al., 2014). As most experiments on non-human primate model of acquired immunodeficiency have shown, HIV/simian immunodeficiency virus (SIV) induces an ongoing activation of the mucosal immune response and a rapid and massive destruction of CD4+T lymphocytes belonging to the GALT system. The cells most affected are the Th17/22 CD4^+^T intestinal subsets involved in the maintenance of the gut barrier (Veazey et al., 1998; Guadalupe et al., 2003; Brenchley et al., 2004, 2006; McGowan et al., 2004; Kim et al., 2013). Thus, HIV reconfigures the intestinal barrier early during the infection through immune mechanisms which promote gut alterations, chronic inflammation, and immunodeficiency.

Human immunodeficiency virus-associated intestinal lesions (“HIV enteropathy”) initially arise due to the imbalance of the epithelial barrier turnover (Sankaran et al., 2008). In time, the increasing inflammation and intestinal permeability contribute to HIV enteropathy and persist despite antiretroviral therapy (ART) (Olsson et al., 2000; Guadalupe et al., 2003; Neff et al., 2018). The histopathological lesions described in this setting involve intestinal inflammation and mucosal atrophy that nevertheless differ from changes recorded in other intestinal immune-mediated diseases (Magro et al., 2013). On a molecular level, animal studies showed that during HIV enteropathy, IECs display a downregulation of host genes and of miRNAs associated with the homeostasis of the epithelial barrier (Sankaran et al., 2005), epithelial permeability (miRNA-21,-130a,-212) (Gaulke et al., 2014; Zhang et al., 2015; Kumar V. et al., 2018), inflammatory signaling (miR-150) (Kumar et al., 2016), and immune activation syndrome (miR-34a) (Mohan et al., 2015). Some of these miRNAs also regulate the gut microbiota (Johnston et al., 2018) or inflammatory pathways associated with irritable bowel syndrome (Zhou et al., 2010, 2015; Fourie et al., 2014). A study performed on the intestinal lamina propria and leukocytes of SIV-infected macaques indicated that the host miRNA gut profile displays a bitemporal variation initially dictated by the rapid reduction of SIV replication and T-cell activation and afterwards correlated with the consequent boost of the inflammatory response (Kumar et al., 2016). Under these conditions gut epithelia gradually lose their protective role and permit the translocation of bacterial immunogenic molecules (Chege et al., 2011; Marchetti et al., 2013) or enteropathogens (Zeitz et al., 1998; Nazli et al., 2010). In the presence of an impaired immune response, these translocations recur and re-enforce the systemic inflammatory response activating CD4^+^T cells and HIV replication in a vicious circle (Kotler et al., 1993; Brenchley et al., 2006; Cassol et al., 2010; Dillon et al., 2014). Thus dysbiosis perpetuates enteric and opportunistic infections and aggravates the course of HIV.

Studies on HIV associated dysbiosis are discordant, hindering comparisons, yet available data shows a major depletion of *Bacteroides* in favor of *Proteobacteria* (Dillon et al., 2014), the enrichment of pathobionts (*Enterococcus, Streptococcus, Staphylococcus, Salmonella*, and *Escherichia* species) and the significant reduction of symbionts throughout the entire intestinal tract (Nishitsuji et al., 2017). Whereas the higher population of *Enterobacteriaceae* increases the risk of translocations, epithelial cells exposed to *E. coli* could increase the susceptibility of CD4^+^Th17 and Th-1 lymphocytes to HIV and viral replication (Dillon et al., 2012).

Intestinal dysbiosis occurs early during HIV and is aggravated by ART itself (Ling et al., 2016; Pinto-Cardoso et al., 2017; also reviewed in Pinto-Cardoso et al., 2018). Hence, patients with immunologic failure on ART, predominantly host species of *Enterobacteriaceae* instead of *Lactobacillus* known for its immunomodulatory and anti-inflammatory activity (Merlini et al., 2011). Otherwise, butyrate synthesized by dominant species in HIV patients (*Fusobacterium nucleatum, Clostridium cochlearium*, and *Eubacterium multiforme*) could reactivate both unintegrated HIV-1 genomes and latent HIV proviruses through HDAC inhibition along with other transcription factors and concurrently explains HIV immunosuppression during ART (Kantor et al., 2009; Imai et al., 2012; Lee et al., 2018). The role of SCFAs in the regulation of mucosal immunity during HIV is controversial due to their implication in the reactivation of various infections as well as in the attenuation of mucosal inflammation and microbial translocation (Das et al., 2015; Ye and Karn, 2015; Dillon et al., 2017). Consequently, certain authors proposed the use of chemical HDAC inhibitors but not of SCFAs along with ART for a successful antiretroviral treatment of HIV reservoirs as well as for the attenuation of the LPS-induced inflammatory response (Archin et al., 2009, 2012; also reviewed in Shirakawa et al., 2013; McManamy et al., 2014).

In conclusion, HIV pathogenesis involves the loss of the immunomodulatory gastrointestinal activity, bacterial translocations and intestinal dysbiosis, independent of ART. Current therapeutic strategies are modest and based on few studies in humans or macaques: the administration of probiotics (Hummelen et al., 2011; Ortiz et al., 2016; also reviewed in Hummelen et al., 2010; D’Angelo et al., 2017; Kazemi et al., 2018), anti-inflammatory drugs (reviewed in Deeks et al., 2013), FMT (Hensley-McBain et al., 2016; Vujkovic-Cvijin et al., 2017) stimulating the immune response with a T lymphocyte-adjuvated-DNA vaccine (Fuller et al., 2012), or using an oral recombinant vaccine with *Cl. perfringens* expressing HIV-1 Gag protein (Pegu et al., 2011).

## Conclusion

The microbiota, the intestinal epithelia and mucosal immunity form an anti-infectious barrier rigorously regulated through complex mechanisms. The host-microbiota-pathogen interactions employ numerous cell receptors and molecules with antibacterial and anti-inflammatory roles, which also modulate the epigenetic and immune response. Together with epithelial and immune cells, these signaling molecules form a network that is essential for intestinal homeostasis and anti-infectious defense. Dysbiosis renders these defense mechanisms non-functional. It consequently aggravates gastrointestinal infections, favors bacterial and LPS translocations in sepsis, unbalances the immune defense in hepatitis viruses and HIV replications and controls the progression of infectious diseases to an unknown extent. A better knowledge on the interactions driving the antimicrobial response of the intestinal barrier is therefore crucial to improve the current anti-infectious armamentarium.

## Author Contributions

Both authors contributed equally to the acquisition, analysis, and critical revision of the manuscript, gave their consent for the publication, and agreed to be responsible for the accuracy and integrity of the manuscript.

## Conflict of Interest Statement

The authors declare that the research was conducted in the absence of any commercial or financial relationships that could be construed as a potential conflict of interest.
